# Autoimmune bullous disorder flares following severe acute respiratory syndrome coronavirus 2 vaccination: a case series

**DOI:** 10.1186/s13256-023-04146-y

**Published:** 2023-09-26

**Authors:** Cody J. Rasner, Brittney Schultz, Kimberly Bohjanen, David R. Pearson

**Affiliations:** 1 University of Minnesota Medical School, Minneapolis, MN USA; 2 Department of Dermatology, University of Minnesota, 516 Delaware St SE, MMC 98, Minneapolis, MN 55455 USA

**Keywords:** Bullous pemphigoid, Pemphigus, COVID, SARS-CoV-2, Vaccination, Autoimmune, Case report

## Abstract

**Background:**

Autoimmune bullous disorders develop due to autoantibodies targeting intercellular adhesion proteins of hemidesmosomes and desmosomes and may be triggered by viral infections and vaccines. Recent reports suggest that the coronavirus disease 2019 vaccination may trigger flares or exacerbations of preexisting autoimmune diseases, including new onset autoimmune bullous disorders. There are less data on whether vaccination against severe acute respiratory syndrome coronavirus 2 may also exacerbate preexisting autoimmune bullous disorders.

**Case presentation:**

Here we present three cases, two white males (ages 69 years and 88 years) with bullous pemphigoid and one white 50-year-old female with pemphigus foliaceus, wherein all individuals developed minor, tractable flares of their preexisting autoimmune bullous disorders after receiving the coronavirus disease 2019 vaccination, which were readily treatable with topical or low-dose systemic corticosteroids.

**Conclusions:**

Dermatologists managing patients with autoimmune bullous disorders should be cognizant of the uncommon potential for flares of the disorder following vaccination for severe acute respiratory syndrome coronavirus 2. Flares of bullous pemphigoid and pemphigus foliaceus following vaccination for severe acute respiratory syndrome coronavirus 2 in these cases were mild and tractable.

## Background

Autoimmune bullous disorders (ABD), including bullous pemphigoid (BP) and pemphigus, arise from autoantibodies directed against hemidesmosomes and desmosomes, respectively. [1] BP, the most common ABD, demonstrates increased incidence with age and results in extremely pruritic urticarial papules and plaques that develop tense vesicles and bullae due to the subepidermal location of vesiculation [1]. The two most common forms of pemphigus are pemphigus vulgaris (PV) and pemphigus foliaceus (PF). Since blisters in pemphigus occur within the epidermis, these disorders are characterized by flaccid blisters, erosions, and crusting. PV affects the mucosae and may affect the skin, while PF is limited to cutaneous surfaces [2]. Suspected diagnosis of BP or pemphigus may be supported by routine histology, direct immunofluorescence (DIF), and via serologic detection of autoantibodies with an enzyme-linked immunosorbent assay (ELISA) and indirect immunofluorescence (IIF); in the latter, titers and antibody levels may correspond to disease severity [2, 3].

In addition to spontaneous development, BP and PF may be triggered by viral infections and vaccines. Infection by human herpesviruses, hepatitis B, and HIV, as well as vaccination against influenza, swine flu, tetanus toxoid, and herpes zoster have been implicated in infantile and adult BP and pemphigus [4–8]. In the current pandemic, there are reports that coronavirus disease 2019 (COVID-19) may exacerbate or even precipitate autoimmunity, including Guillain–Barré syndrome and systemic lupus erythematosus [4, 5, 9, 10]. Various additional cutaneous reactions following administration of the COVID-19 vaccine have been reported, including urticarial and morbilliform eruptions, pseudo-chilblains, vesicular eruptions, and others. Furthermore, there have been reports of the development of new-onset ABD after receipt of both the Pfizer-BioNTech BNT162b2 mRNA COVID-19 vaccine and Moderna mRNA-1273 COVID-19 vaccine [6, 11]. A recent multicenter study conducted in Italy revealed that vaccination was associated with the emergence of bullous pemphigoid (BP) in 21 patients. Although these cases presented clinically similar to idiopathic BP, the study highlighted a notable male predominance and a diminished humoral response to BP230, suggesting a possible distinct disease mechanism within this subset of vaccine-related BP cases [12]. An important consideration is thus whether vaccination against severe acute respiratory syndrome coronavirus 2 (SARS-CoV-2) may also exacerbate preexisting ABD, and the degree of severity of such flares. Here we discuss three cases, two of BP and one of PF, wherein individuals developed minor, tractable flares of their preexisting ABD after receiving the COVID-19 vaccination.

## Case presentation

### Case 1

An 88-year-old white male with history of BP presented with diffuse pruritus approximately 24 h after receiving the second dose of the Pfizer-BioNTech BNT162b2 mRNA COVID-19 vaccine. He was diagnosed in 2016 by IIF with a positive ELISA for BP230 and was moderately well controlled on 5–10 mg of prednisone daily and topical triamcinolone acetonide 0.1% cream as needed. He had previously declined treatment with steroid-sparing immunosuppressive agents including mycophenolate mofetil and methotrexate. Since the onset of the pandemic, the patient had never displayed symptoms concerning for COVID-19 nor been tested.

**Fig. 1:**
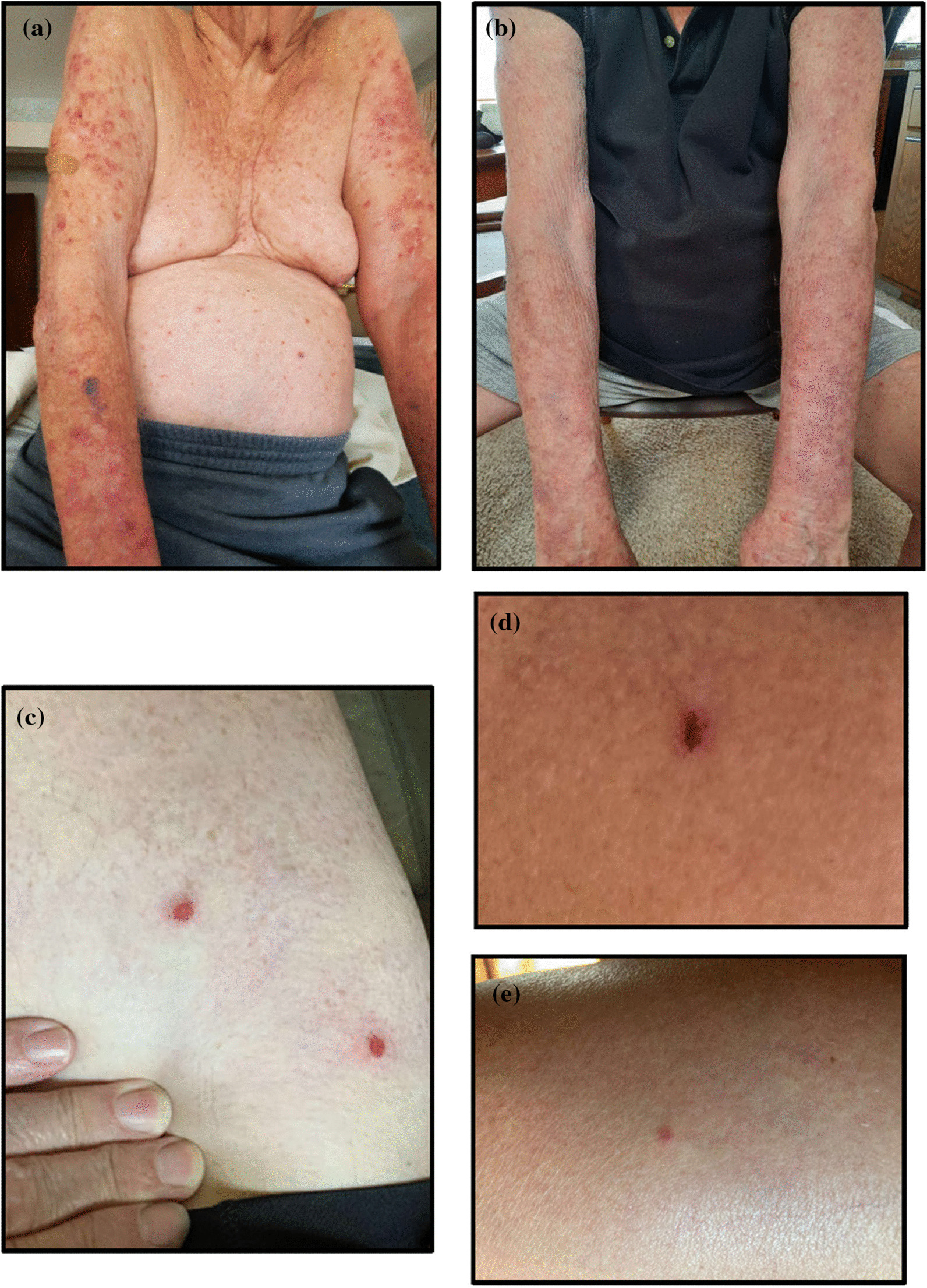
Blistering disease in the context of COVID-19 vaccination. Erythematous, urticarial papules and confluent plaques with admixed vesicles and erosions on the torso and extremities before treatment (**a**) and 5 weeks after treatment (**b**) in case 1. Widely scattered crusted erosions and pink, scaly papules over upper thigh (**c**) in case 2. Superficial, crusted erosions on the posterior neck before treatment (**d**) and 6 weeks after treatment (**e**) in case 3

Examination demonstrated numerous erythematous, urticarial papules and confluent plaques on the torso and extremities, with admixed tense vesicles and erosions (Fig. 1a).

After discussion of treatment options, the patient was prescribed an oral prednisone taper, starting at 40 mg and reduced by 10 mg weekly, until returning to his baseline 5–10 mg daily. After 5 weeks, his pruritus completely resolved, and his skin lesions were well healed (Fig. 1b).

### Case 2

A 69-year-old white male with a history of BP and erythrodermic psoriasis presented with scattered erosions 2 weeks after receiving his second dose of the Moderna mRNA-1273 COVID-19 vaccine. Initial diagnosis of BP was in late 2018 by histopathology, including consistent DIF, and was later confirmed with consistent IIF and ELISA for both BP180 and BP230 in early 2019. Due to concomitant erythrodermic psoriasis, he had been initially managed with cyclosporine 3 mg/kg divided into two daily doses and then transitioned to adalimumab 40 mg subcutaneous every other week for maintenance treatment in May 2019. He was not on long-term treatment specifically directed against BP. Of note, the patient had symptomatic COVID-19 4 months prior to vaccination, confirmed by a positive SARS-CoV-2 PCR nasal swab.

On examination, he had few, widely scattered crusted erosions and pink, scaly papules on his extremities (Fig. 1c).

The patient’s symptoms gradually improved with application of augmented betamethasone dipropionate 0.05% ointment two to three times weekly; no additional intervention was required. During this time, the patient continued with adalimumab injections.

### Case 3

A 50-year-old white female with a history of PF presented with a few itchy, crusted erosions 1 week after receiving her first dose of the Pfizer-BioNTech BNT162b2 mRNA COVID-19 vaccine, which increased slightly following her second vaccination. Vaccines were administered in March 2021 and April 2021, respectively. Initial diagnosis of PF was confirmed by histopathology, including consistent DIF, in 2010. She was first managed with prednisone due to intolerance of azathioprine and mycophenolate mofetil. In 2019, she demonstrated a positive IIF for cell surface immunoglobulin G4 (IgG4) on monkey esophagus (titer > 1:40) and intact human skin (titer > 1:40), and positive desmoglein-1 autoantibodies (84 units; positive > 20 units), and was subsequently treated with rituximab 1000 mg every 2 weeks for two doses in June 2019 with an excellent clinical and serologic response; erosions resolved and her IIF and ELISA were negative. Following discontinuation of prednisone in October 2019, she was maintained off treatment.

Examination revealed four superficial, crusted erosions on the posterior neck, torso, and upper extremities (Fig. 1d and e). Repeat IIF for cell surface IgG4 was negative on monkey esophagus and borderline on intact human skin (titer 1:10); desmoglein-1 autoantibodies were slightly elevated (23 units; positive > 20 units).

The patient started application of augmented betamethasone dipropionate 0.05% ointment but eventually required a low-dose oral prednisone taper (10 mg and reduced by 2.5 mg weekly) due to persistent erosions. Her symptoms returned to baseline by her follow-up visit 10 weeks later. Of note, patient 3 did have a successful immune response after vaccination as demonstrated by a positive spike receptor-binding domain (RBD) protein antibody titer.

## Discussion

ABD may precipitate from a variety of mediators that are often challenging to definitively pinpoint [7, 8]. Given the dysregulation of the immune system in BP and PF, there is concern for the potential to exacerbate disease activity after inducing an immune response with administration of the SARS-CoV-2 vaccine. In a single-institution academic dermatology clinic, we identified three patients with ABD who flared after receiving the COVID vaccine (Table 1). Of the cases identified, two opted to use systemic steroids, while topical steroid application was sufficient for the remaining patient. All three patients significantly improved or returned to baseline by their follow-up appointments ranging between 5 weeks and 10 weeks after the initial visit.

**Table 1:** Baseline characteristics, flare onset latency, serologic results, and outcomes for three cases of flares of preexisting ABD following COVID-19 vaccination

	Age/sex/ethnicity Diagnosis/vaccine	Onset latency (dose #)	IIF/ELISA results at diagnosis (date)^a^	Baseline treatment	IIF/ELISA results with current flare^a^	Recent treatment	Outcome	SARS-CoV-2 infection status
Case 1	88/M/W/BP/Pfizer	24 h (dose 2)	ELISA (2020): BP180, negative; BP230 15.8 units	Prednisone 5–10 mg daily, triamcinolone 0.1% cream	ELISA: BP180, negative; BP230 10 units IIF: 1:10, 240 BMZ IgG (ME), 1:2,560 (roof, HSS)	Prednisone 40 mg daily taper	Baseline by 5 weeks	Negative
Case 2	69/M/W/BP/Moderna	14 days (dose 2)	IIF (2019): > 1:40,960 BMZ IgG (ME), 1:10,240 (roof, HSS) ELISA (2019); BP180, 15 units; BP230, 63 units	None (prior cyclosporine; on adalimumab for psoriasis)	Not available	Augmented betamethasone 0.05% ointment	Resolved by 6 weeks	Positive (confirmed by SARS-CoV-2 PCR 4 months prior to vaccination)
Case 3	50/F/W/PF/Pfizer	7 days (dose 1)	IIF (2019): > 1:40 cell surface IgG4 (ME and IHS) ELISA (2019): Dsg-1, 84 units	None (prior prednisone and rituximab, last administered June 2019)	IIF: negative (ME), 1:10 cell surface IgG4 (IHS) ELISA: Dsg-1, 23 units	Prednisone 10 mg daily taper, augmented betamethasone 0.05% ointment	Baseline by 10 weeks	Negative

ABD, autoimmune bullous disorders; BMZ, basement membrane zone; BP, bullous pemphigoid; Dsg-1, desmoglein-1; ELISA, enzyme-linked immunosorbent assay; F, female; IHS, intact human skin; IIF, indirect immunofluorescence; HSS, human split skin; M, male; ME, monkey esophagus; Moderna, Moderna mRNA-1273 COVID-19 vaccine; PCR, polymerase chain reaction; Pfizer, Pfizer-BioNTech BNT162b2 mRNA COVID-19 vaccine; W, white

^a^Reference ranges: IIF: positive: > 1:10 (ME, IHS, HSS); borderline/indeterminate: 1:10 BMZ IgG (ME, HSS), 1:10 cell surface IgG4 (ME and IHS); negative: < 1:10 (ME, HIS, HSS); ELISA: BP180 and BP230: > 9 units; Dsg-1: > 20 units; borderline/indeterminate: Dsg-1: 9-20 units; negative: Dsg-1: < 9 units.

Subsequently, using a deidentified medical record search query with ICD codes L10.X, L12.X, and L13.X, we identified 160 ± 3 patients (the search query program outputs inexact numbers to maintain confidentiality in cases of rare disease that may make the data identifiable) who were seen in our dermatology clinics between 1 January 2021 and 20 July 2021; no additional cases of ABD flare after vaccination were identified. Individual data, including the fraction of 160 ± 3 patients who received COVID-19 vaccination, are unknown.

Identifying a potential causal relationship between vaccination and disease flare is important for appropriately assessing risk and counseling patients. Notably, in case 3, a potential relapse in disease due to discontinuation of rituximab is less likely given that it was stopped in June 2019, while her PF flare occurred shortly after vaccination in March of 2021. This observation supports the hypothesis that her increased PF activity was vaccine related, highlighting a potential association between the vaccine and the perturbation of disease. This study was also limited by the timeline of antibody titer testing, as disease activity levels may have measured higher if antibody titers were tested closer to onset of symptoms rather than during or after treatment of the flare. Importantly, all cases of pemphigoid and pemphigus eruptions following vaccination were readily treated and patients improved without the need for prolonged changes in baseline medications. Prompt amelioration of symptoms suggests the unintended vaccine response is mild and transient and is corroborated by low IIF and disease-specific ELISA antibody titers in two of the cases.

The precise mechanism by which the vaccine may have exacerbated BP or PF in these patients has not been elucidated. It is possible that the intentional immune response triggered to induce immunity to SARS-CoV-2 may also upregulate inflammatory mediators known to be elevated during an acute COVID-19 illness, including interleukin (IL)-1β, IL-2, IL-6, IL-8, IL-10, IL-17, IL-18, CXCL10, and CCL2, leading to changes in T-regulatory (CD4+ , CD25+ , FOXP3) cells [13]. Of these inflammatory molecules, IL-1β, IL-6, IL-8, and IL-10 are also upregulated in ABD [1]. A previous study by Solimani *et al*. demonstrates no significant change in autoantibody titers following booster vaccination for SARS-CoV-2, though whether or not the patients had a flare of their disease was not reported [14]. Such widespread activation of the immune system, coupled with activation of overlapping inflammatory pathways, may provoke preexisting ABD through increased autoantibody production. Recent reports have described flares of hereditary bullous disorders and acquired ABD following the administration of the COVID-19 vaccination [15–17]. Alternatively, evidence suggests that anti-SARS-CoV-2 antibodies do not directly cross-react pemphigus or pemphigoid autoantibodies [18], though whether SARS-CoV-2 infection indirectly augments the immune system, therein precipitating ABD and other autoimmunity, remains unclear. This study is therefore inherently limited by the lack of laboratory data for these patients, as it pertains to autoantibody titer levels during their disease flare. Further studies are needed to elucidate autoantibody titer changes in the context of possible vaccine-related ABD activity. Importantly, the potential adverse event following immunization (AEFI) should be described as rare and tractable, and clinicians may safely and confidently encourage patients to receive COVID-19 vaccination [19].

## Conclusions

We present three cases of mild flares of preexisting ABD occurring shortly after receiving the COVID-19 vaccination. Clinicians should counsel patients as to the possible unintended effects of vaccinating for SARS-CoV-2 and how these effects may exacerbate preexisting BP and PF. Individuals with preexisting ABD and other autoimmune disease have been cautiously considered since the beginning of the pandemic, and the evidence presented here highlights the treatability of any AEFI resulting from the COVID-19 vaccine for this subpopulation of people with autoimmune disease.

## Data Availability

The data for this case report are located at University of Minnesota and Fairview Hospital, Minneapolis, Minnesota, USA.

## Abbreviations

AEFI: Adverse event following immunization
ABD: Autoimmune bullous disorder
BP: Bullous pemphigoid
DIF: Direct immunofluorescence
ELISA: Enzyme-linked immunosorbent assay
IIF: Indirect immunofluorescence
PF: Pemphigus foliaceus
PV: Pemphigus vulgaris
RBD: Receptor-binding domain
WHO: World Health Organization
